# Role of the World Organisation for Animal Health in global wildlife disease surveillance

**DOI:** 10.3389/fvets.2024.1269530

**Published:** 2024-03-20

**Authors:** Lesa Thompson, Claire Cayol, Lina Awada, Sophie Muset, Dharmaveer Shetty, Jingwen Wang, Paolo Tizzani

**Affiliations:** ^1^Regional Representation for Asia and the Pacific, World Organisation for Animal Health, Tokyo, Japan; ^2^Preparedness & Resilience Department, World Organisation for Animal Health, Paris, France; ^3^Data Integration Department, World Organisation for Animal Health, Paris, France; ^4^World Animal Health Information and Analysis Department, World Organisation for Animal Health, Paris, France

**Keywords:** disease reporting, disease surveillance, One Health, wildlife health, Wildlife Health Framework, WOAH

## Abstract

This paper examines the role of the World Organisation for Animal Health (WOAH) in the global surveillance and management of pathogens. Since the creation of WOAH, one of its missions has been to ensure transparency of the global animal health situation. WOAH established a Working Group on Wildlife in 1994 to inform and advise WOAH Members, leadership, and technical teams on issues relating to wildlife health. In 2020 it conducted a consultation with its Members before developing a Wildlife Health Framework to improve global health and wildlife conservation. WOAH Members report diseases in wildlife, but detections are dependent on the surveillance systems in place. As an example of data collected in the most recent years (2019–2023), 154 countries have reported 68,862,973 cases, through alert messages and weekly updates, for 84 diseases. One-hundred and fifty countries have reported 68,672,115 cases in domestic animals and 95 countries have reported 190,858 cases in wild animals. These figures illustrate the performance of the organization in collecting data on wildlife, and provide an indication of the difference in completeness of data collected in domestic animals and wildlife. There are several challenges to wildlife disease surveillance and real figures remain unknown; they depend on the existence, quality and sensitivity of national surveillance. A WOAH-led One Health approach with cross-sectoral collaboration is needed to improve surveillance sensitivity, address the challenges and help safeguard wildlife population health and biodiversity conservation.

## Introduction

Historical and recent disease threats to animals and humans worldwide have highlighted the need to consider diseases in a global context, as multi-host pathogens do not recognize the boundaries between species or countries. Initially founded in 1924 in response to the international spread of rinderpest, considered the deadliest cattle disease in history, the World Organisation for Animal Health (WOAH, founded as OIE) is an intergovernmental organization with a mission to improve animal health worldwide (1, 2). To this end, WOAH is now the global authority on animal health and focuses, among other objectives, on transparent dissemination of information on prioritized animal diseases (3).

Although WOAH is best known for its work with veterinary authorities on farmed animal diseases, Members of the organization recognize the importance of taking a holistic approach when addressing transboundary animal disease management. Wildlife health has been considered by WOAH and its Members from as early as 1954. In 1994, a Working Group on Wildlife was established to inform and advise WOAH Members, provide leadership, and technical input on issues related to wild animal health (captive, feral or free-ranging). Additionally, nearly all 183 WOAH Members have adopted the approach of nominating a Focal Point for Wildlife – forming a global network responsible for collecting and reporting disease information in wildlife to WOAH. Members have agreed that impact on wildlife is considered in the criteria for listing diseases by WOAH, and that information sharing on wildlife is considered within the mandatory scope of most diseases listed by WOAH (4, 5).

Several diseases have crossed interfaces between humans, livestock and wildlife, and are transboundary between countries. Wildlife and domestic livestock have been affected by shared diseases such as African swine fever, lumpy skin disease or avian influenza (6–11). Wildlife may also be important in the epidemiology of zoonotic diseases, for example, Nipah virus (12). Avian influenza that circulates widely in wildlife (mainly as low pathogenic avian influenza) and has the potential to become pathogenic to people, usually requiring a domestic animal intermediate host (13). Early disease detection and information sharing enable better risk management of disease transmission within populations and spillover to other species (including humans), often with significant financial benefits (14, 15). Acknowledging the importance of disease surveillance in wildlife, WOAH Members committed to report detection of diseases listed by WOAH in wild animals, through the World Animal Health Information System (WAHIS) (16); other reporting channels and modalities are also currently under review.

**Figure 1:**
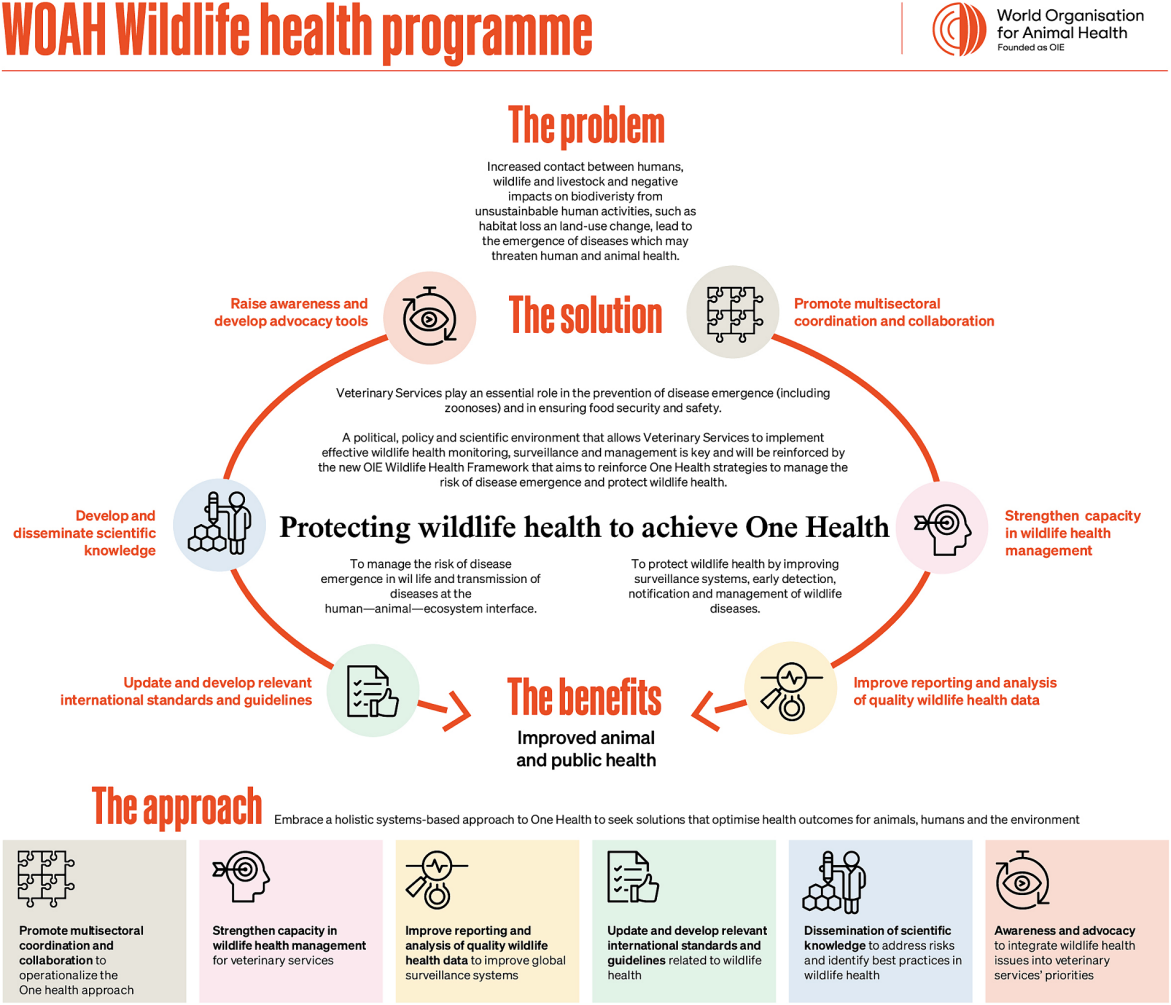
The WOAH Wildlife Health Framework was developed to ensure that wildlife health issues are fully integrated and transversally addressed in WOAH’s core work such as Standards and guidelines, Performance of Veterinary Services (PVS) pathway, disease notification systems, among others to better support WOAH Members. Domestic animals have been at the center of animal health strategies worldwide, but equal investment in wild animal health is needed to ensure a holistic approach to animal health management, maintain healthy animal populations (both wild and domestic), ensure healthy ecosystems, and contribute to global health.

In 2020, in light of lessons learned from the COVID-19 pandemic, WOAH launched an extensive stakeholders’ consultation, leading to the development of a comprehensive Wildlife Health Framework (Figure 1) dedicated to the protection of wildlife health within the One Health context (17). WOAH’s historical, present, and prospective future contributions to wildlife disease surveillance are described in this article, to clarify and raise awareness of the organization’s role in supporting and sharing information on global wildlife disease surveillance.

## Past and current role of WOAH in wildlife disease surveillance

For Members in need of support, WOAH provides guidelines and standards related to animal diseases to establish national “surveillance,” which is defined as “the systematic ongoing collection, collation, and analysis of information related to animal health and the timely dissemination of information so that action can be taken” (5, 18).

At its creation, Members mandated WOAH, among other things, to promote research concerning the contagious diseases of livestock for which international collaboration is deemed desirable. As early as 1952, WOAH (then known as the OIE) recommended more research on the wild reservoirs of relevant livestock species diseases. In 1954, the first resolution of the World Assembly of Members on wildlife was adopted (19). In 1965, WOAH highlighted the need for research on bat rabies to safeguard sustainable bat populations while also protecting public health. The need to preserve biological conservation was also raised by WOAH at the joint OIE/ONS/FAO 1967 meeting (20). The establishment of an *ad hoc* group for Wildlife in 1993—which rapidly transformed into the permanent WOAH Working Group on Wildlife in 1994—was a logical concretization of the involvement of WOAH and its Members in the global discussion on wildlife diseases.

At the request of countries, the world-renowned experts of the WOAH Working Group on Wildlife have prepared recommendations and statements, and overseen numerous scientific publications on the surveillance and control of the most important wildlife diseases, while providing technical guidance to manage outbreaks in wild animals for almost three decades. Since 2010, WOAH’s action on wildlife health has been organized around the network of Focal Points for Wildlife who undertake professional training on wildlife health surveillance-related topics every 2 years. These Focal Points are generally civil servants, working for the Ministry of Agriculture or Environment (or equivalent); they are responsible for establishing and maintaining national networks of wildlife experts and for submitting wildlife disease information to WOAH. Additionally, WOAH Reference Laboratories are designated to pursue scientific and technical problems for specific diseases, and WOAH Collaborating Centers provide expertise and support, and promote international collaboration for specific topics (21, 22). Several of these Reference Laboratories and Collaborating Centers have experts in topics related to wildlife health (e.g., Collaborating Center on Research, Diagnosis and Surveillance of Wildlife Pathogens (associate) in the USA and Canada, Collaborating Center on Training in Integrated Livestock and Wildlife Health and Management in South Africa, and the Collaborating Center on Wildlife Health Risk Management in Australia).

To take a step further in achieving its mandate, WOAH developed a Wildlife Health Framework – this is WOAH’s Global Strategy for Wildlife Health. As part of its 2020 early design phase, a stakeholders’ consultation showed that 95% of WOAH Members considered that Veterinary Services should be involved in the epidemiological surveillance of diseases in wildlife at the human–animal–ecosystem interface (23). Iterative contributions from the Working Group on Wildlife, the stakeholders’ consultation, WOAH staff worldwide and external partners were used to prepare the WOAH Wildlife Health Framework. This document aligns the WOAH 7th Strategic Plan (2021–2025) which includes consideration of intersectoral issues such as the role of wildlife in disease emergence and spread, and works toward integrating wildlife health into all areas of the organization’s activity (24). The two objectives in the framework aim to support Members to improve (i) their ability to reduce, anticipate and manage the risk of pathogen emergence and transmission at the human–animal–ecosystem interface, and (ii) early detection, notification and management of wildlife diseases. The Framework was thus designed with a dual goal of improving global health and wildlife conservation.

A key output of the Framework is “improved quality data [on wildlife health events and potential drivers, especially wildlife trade] collection, reporting, analysis and use.” As such, WOAH supports Members, particularly their Veterinary Services, to improve health event surveillance and reporting, as described below. Specifically, activities under the Framework aim to support WOAH Members to improve their ability to manage the risk of health event occurrence (including pathogens) in wildlife and transmission at the human-animal-ecosystem interface, while considering the protection of wildlife. The Framework recognizes the costs associated with appropriate wildlife health surveillance systems, but also highlights that the costs and risks to public health and animal health of not investing are greater. Additionally, Members receive technical and structural support from WOAH and its network of Reference Laboratories and Collaborating Centers to improve surveillance systems, early detection, notification and management of wildlife health events. The Framework has been converted into an action-oriented program with a 5-year implementation plan. In 2021, at the 88th General Session, the World Assembly of WOAH Delegates adopted Resolution No. 31 on “How WOAH can support Veterinary Services to achieve One Health resilience” (25). This Resolution further recognizes the key role of wild animals in global disease management and strengthens the inclusion of wildlife health in the organization’s work.

The stocktaking and baseline assessment of the Wildlife Health Framework consisted in a set of consultations and surveys (in 2022 and 2023) to better understand country-level surveillance as well as Members’ capacity, needs and challenges for wildlife disease surveillance. The most challenging task identified by Focal Points for Wildlife to fulfill their role was the integration of wildlife health into national animal health strategies. A survey revealed that Veterinary Services were involved either alone (43% of respondents) or in association with other sectors (43% of respondents) in management of wildlife health events (26). However, important needs regarding investigation of wildlife outbreaks were highlighted, with 63% of Members reporting impediments to collecting, handling or transporting wildlife samples (26). The stocktaking step also revealed that a high level of wildlife health disease recording (69%) used unreliable recording systems (paper records or local computer recording systems); this underscores the need for reinforced capacity on wildlife health information management (27).

**Figure 2:**
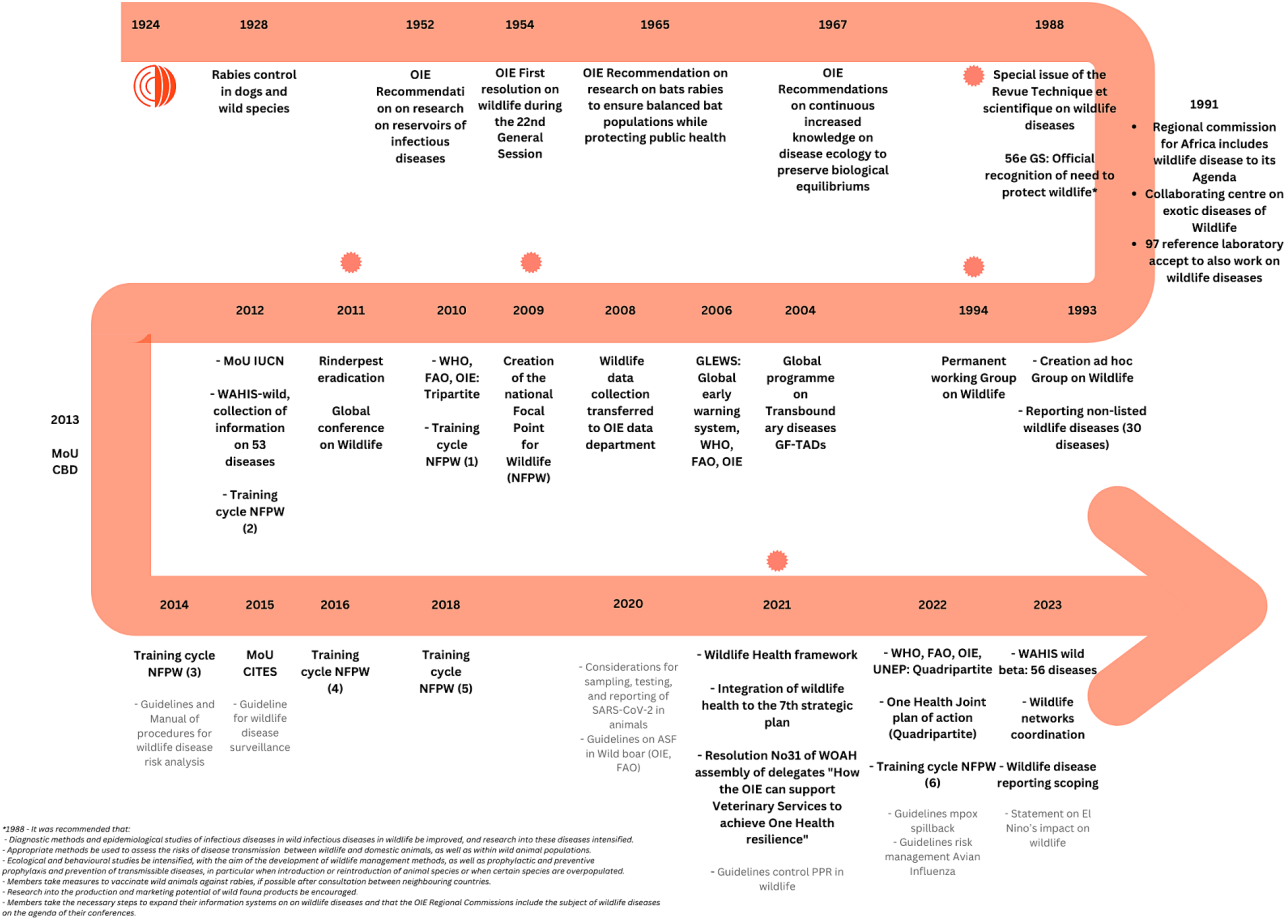
Timeline showing WOAH’s evolving role in data collection, surveillance and reporting of wildlife diseases since 1924 and as of 2023.

The steps of WOAH and the evolution of its role in data collection, surveillance and reporting of wildlife diseases are summarized in Figure 2.

Indeed, when it comes to animal health data reporting, WOAH has developed processes to organize reporting priorities with its Members and experts, allowing consensus and engagement. WOAH provides a common global tool for Members to report both livestock and wildlife health events – the World Animal Health Information System (WAHIS) (28). To support data reporting from Members, WOAH shares definitions and standards, as well as guidelines, and conducts regional trainings or one-on-one sessions, as needed, ensuring useful and accurate reporting. WOAH Standards include disease and diagnostic test definitions (5, 18, 29, 30). In addition, WOAH conducts “web scraping “of disease signals using event-based surveillance tools (web-based systems that allow detection and collection of relevant news on diseases based on pre-determined search algorithms), to coordinate with Members and support them in their reporting obligations. WOAH also works closely with global and regional partners from various sectors to share information on disease events at the human-animal-ecosystem interface, and to support Members in their disease surveillance, early warning and preparedness efforts. Despite these efforts, engaging WOAH Members in voluntary wildlife disease reporting was deemed challenging for various reasons such as the lack of information related to wildlife health reaching WOAH Members, a non-fit for purpose reporting system, and wildlife health often not falling under the Veterinary Services’s realm of action. After identifying the source of the challenges, WOAH has taken steps to set up corrective actions and is currently working on a new initiative regarding early warning and enhanced information on these non-WOAH-listed diseases that will be better adapted to the needs of WOAH Members and their partners working with wildlife. Meanwhile, an interim system is in place to collect information on non-WOAH-listed diseases on a voluntary basis.

To support improved capacities for wildlife surveillance in line with the Framework, WOAH is embracing the One Health approach and integrating wildlife health more fully into existing established programs such as the Performance of Veterinary Services (PVS) Pathway evaluations and Laboratory Twinning activities (31). An example of a Laboratory Twinning activity is represented by the US-Thailand Wildlife Health Twinning Project, based on expertise sharing on wildlife disease risk assessment and improvement of the national wildlife disease surveillance system (32). Online e-learning materials relevant to wildlife health are being developed based on WOAH Standards and Guidelines, and digital or printable communication tools are also made available (see, e.g., Wildlife health is everyone’s health). In addition, all material developed by WOAH for training its Focal Points for wildlife is made available online (33). The International Health Regulations (IHR) – PVS National Bridging Workshops (NBWs) assist countries to prepare for and respond to prioritized health threats, and have begun to involve more participants from environmental sectors including wildlife health representatives (34). WOAH is also working with other global partners on initiatives which strive to promote and normalize multi-sectoral collaborations on health issues, such as the Nature for Health Initiative (35). In this context, it is worth highlighting the recent integration (March 2022) of the United Nations Environment Program (UNEP) into the Tripartite collaboration (comprising the Food and Agriculture Organization of the United Nations (FAO), World Health Organization (WHO) and WOAH) to accelerate a coordinated strategy on human, animal and ecosystem health. With this, the Tripartite has formally become the Quadripartite. The work of the newly expanded alliance is focused on the One Health Joint Plan of Action (OH JPA), with six main action tracks: (i) enhancing countries’ capacity to strengthen health systems through a One Health approach; (ii) reducing the risks of emerging or re-emerging zoonotic epidemics and pandemics; (iii) controlling and eliminating endemic zoonotic, neglected tropical or vector-borne diseases; (iv) strengthening food safety risk assessment, management and communication; (v) stemming the silent pandemic of antimicrobial resistance (AMR); and (vi) better integrating the environment into the One Health approach (36).

## Wildlife disease reporting to WOAH

Since the creation of WOAH, one of its main missions has been to ensure transparency of the global animal health situation and improve knowledge of animal diseases, including those transmissible to humans (i.e., zoonoses). To this purpose, WOAH Members have committed and are required to notify relevant information on their animal health situation in domestic animals and, when relevant, in wildlife, in compliance with the provisions of WOAH’s Terrestrial Animal Health Code (Terrestrial Code) and Aquatic Animal Health Code (Aquatic Code) (37, 38). This has two main objectives: (i) sharing information on the known situations of diseases prioritized by WOAH Members (called “listed diseases”) and (ii) early information sharing on unusual animal health events both for such priority listed diseases and for emerging diseases. To ensure engagement of countries, the criteria for disease prioritization, list of prioritized diseases and early information sharing scope are subject to Members’ review and adoption by voting.

Animal disease information submitted from countries to WOAH is verified through an internal process and made publicly available through WAHIS. The system therefore comprises highly specific data (based on validated diagnosis tests and validated by competent authorities and WOAH) (5, 18, 39–41). Wildlife data are collected on about 100 listed diseases from 183 WOAH Members, and a few non-Members. However, countries and territories have different capacities in terms of disease surveillance, detection, and diagnosis. Moreover, test validations for many wild species and diseases may not be available, rendering submission to WAHIS unworkable for some wildlife disease information. In fact, the occurrence of some listed diseases in wild host species does not fit the WOAH Terrestrial Animal Health Code definition and does not fall under mandatory notification. Collecting these data in a standardized and coordinated manner therefore represents one of the main challenges for a global surveillance system.

For each listed disease, countries are requested to provide information through various reporting streams. For unusual events (such as the first occurrence of a disease in a country, or occurrence in an unexpected species), countries are asked to provide an immediate “alert” report, followed by weekly updates until the event is resolved or stabilized. Conversely, for more stable situations (such as when the disease is considered endemic), they should update the situation for each listed disease on a six-monthly basis. Each semester, they should provide at least the disease situation in the country (“presence,” “absence” or “no information collected”). Where possible, countries are asked to complement this with information on surveillance and control measures implemented, and quantitative data on diseases present including number of outbreaks, cases and deaths.

Since the WOAH Working Group on Wildlife was established, the coordinated collection of data on wildlife health has been extended to cover a further 50 or so disease groups (with each group including one or more pathogens) deemed to be a priority by experts, mainly for conservation purposes. To support countries’ notification and clarify reporting boundaries, Technical Disease Cards have been published on the WOAH website (42). Although Members are encouraged to contribute to this additional effort, they are not legally obliged to do so. As alluded to above, data on wildlife diseases are mostly neglected with variable degrees of surveillance systems in place.

In addition, when addressing wildlife diseases, it is important to account for the historical, cultural, political, economic, and sociological context in countries and territories, as the perceived value of wild species might vary depending on these factors (43–45). For this reason, WOAH is placing increased importance on its epidemic intelligence framework - evaluating, assessing and integrating information derived from official data collected from WOAH’s experts & partner network, as well as data from unofficial sources (e.g., using an information system for automatized collection of information such as the Epidemic Intelligence from Open Source (EIOS) initiative) that presents useful sources to assist in better evaluating the real occurrence of disease (46). These complementary sources also support Members in their reporting activities, and in risk assessment and communication. To minimize the number of unreported events, WOAH has been actively searching for non-official information, rumors and signals relating to animal health and veterinary public health events around the world since 2002. As a result of this activity and the incorporation of a web-based system for the automatic detection of relevant news, WOAH is able to review approximately 120,000 news items each year. Consequently, on average, about 10–14% of events reported through immediate “alert” are additionally submitted to WAHIS each year. This value represents the potential of epidemic intelligence activities to increase mandatory reporting. In the past year, special efforts have been made to ensure increased sensitivity for detecting disease events in wildlife, including potential new and emerging threats, through the development of specific search algorithms in several languages.

Despite bias associated with wildlife disease reporting by Members, some figures are provided in this section to illustrate data collected by the system in recent years (since 2019), compared with the same figures for domestic animal diseases. The purpose of these numbers is not to present any in-depth analysis of the information collected by WOAH, but to provide some data for reflection on the role of the organization. Between 1 January 2019 and 2 November 2023, 154 countries reported 68,862,973 cases through alert messages and weekly updates to WAHIS, for 84 different diseases. One-hundred and fifty countries have reported 68,672,115 cases in domestic animals and 95 countries have reported 190,858 cases in wild animals. These simple figures indicate the difference in completeness of data collected by WAHIS for domestic animals and wildlife. The percentage of countries being able to detect and report information on exceptional epidemiological situations through WAHIS is lower for diseases in wildlife than for domestic animals. The diseases for which the highest number of countries reported presence in wildlife through this alert channel were: avian influenza of high pathogenicity (*N* = 78 countries), African swine fever (*N* = 26 countries), SARS-CoV-2 in animals (*N* = 18 countries), rabbit hemorrhagic disease (*N* = 5 countries), and West Nile fever (*N* = 5 countries).

Despite being incomplete, the data collected on diseases in wildlife can be useful for Members and general users. They can provide an idea of the global situation of several diseases, and their evolution in time and space. They can be used for risk assessment, to assist decision-making, and to assess the impact of diseases on biodiversity and conservation. As part of the conservation objective, it is important to present the data collected in context. Indeed, while wildlife can represent a reservoir in certain situations (thereby increasing the risk of transmission of pathogens to livestock and humans), it can also be infected through contamination by livestock and humans. These spillover and spillback phenomena have been widely described in the literature, and it is important to consider wildlife from a health point of view not just as a potential source of disease but also as a potential victim (47–50). WOAH regularly uses this information to produce situation reports on selected diseases to provide easy and “digestible” access to data. This is done regularly for diseases such as African swine fever, highly pathogenic avian influenza and SARS-CoV-2 which are considered relevant for both domestic animals and wildlife (51–53). In addition, a specific situation report on wildlife disease reporting is produced monthly to assess the importance of reported cases in wildlife for animal health, public health and biodiversity conservation (54). Official data reported for aquatic animals (including wildlife) are also periodically presented, acknowledging associated surveillance gaps (55).

## WOAH’s future role in wildlife health

Since its creation, 183 Members have progressively adhered to the principles and rules of WOAH by joining the organization. Driven by the needs expressed by Members and to adapt to the changing global animal health situation, WOAH has continuously built and enriched its activities and contributions to the surveillance and management of diseases and welfare in wild and domestic, terrestrial and aquatic animals. WOAH’s implementation of the Wildlife Health Framework now positions the organization to support its Members worldwide in strengthening their wildlife disease surveillance efforts. WOAH, as an international organization responsible for ensuring transparency on animal diseases and with a well-structured network of veterinary services—is in a unique position to collect information on wildlife disease distribution and surveillance activities at the global level. The scope of this information gathering is gradually evolving, in constant consultation with scientific experts, and, first and foremost, its Members.

To minimize the burden of data collection on Members, WOAH is regularly consulting its 70-plus partner international organizations, some of which are already collecting key data for the global surveillance and epidemic intelligence effort (among them, UNEP, IUCN, CITES, and Interpol). Acknowledging that wildlife disease surveillance is by substance a collaborative and multisectoral activity, WOAH encourages Members to take part or lead national networks of public and private stakeholders collecting wildlife health information in the field (56).

The challenges ahead will most probably include continuing to reflect on the synergies between existing information systems, adopting the most adapted technologies, and making good use of data of all kinds and in all formats in an integrated effort to provide Members with the best possible support in their understanding of the global situation and risks. This will involve not only consideration of technological advances but also workflow and responsibility for reporting.

These efforts will need to address the various challenges and gaps highlighted in this paper. In particular, they will address the relative lack of surveillance and resources dedicated to the detection of diseases in wildlife in many countries, as well as the lack of communication between different health sectors which results in poor information sharing at the international level.

To move toward earlier risk assessment and communication, it is necessary to develop international efforts to monitor and analyze drivers and unusual morbidity/mortality events, as well as non-infectious causes of wildlife mortality in addition to pathogen surveillance. WOAH, other members of the Quadripartite, and international partners in general have a duty to lead by example, by coordinating their exchanges of information for risk analysis and communication more effectively. This process of reflection has already begun and must continue in the years to come (57).

Enhanced monitoring of the implementation of WOAH’s various standards and guidance documents is required to more effectively tailor the guidance to the Members’ needs. The organization has launched a transversal program, the Observatory, that provides an overview of the uptake of international standards by Members. It provides valuable feedback on implementation and effects of Standards, contributing to the progressive improvement of their implementation as well as to the constant assessment of WOAH’s corporate initiatives. The Observatory program will help WOAH adjust its activities to Members’ needs, including those relating to wildlife (58). The development of new guidance or statements on wildlife health will necessarily adapt to topicalities (e.g., Considerations for emergency vaccination of wild birds against high pathogenicity avian influenza in specific situations) but also intensively use foresight (e.g., Early warning and early action – the coming El Niño Southern Oscillation phenomenon and health impacts) (59, 60). Recommendations for increased awareness of wildlife disease and ecosystem balance from WOAH occurred long before wildlife diseases and conservation of biodiversity became topical. Nevertheless, tangible actions were delayed, and more decisive measures and engagement are yet to come - for instance, the incorporation of the One Health principle in its core mission statement and the inclusive definition of animals in its Code and Manual. By identifying and addressing old and new challenges, fostering international collaboration, and embracing a One Health approach in alignment with the Quadripartite collaboration, WOAH will contribute to a safer and more secure future for both animals and humans in the face of evolving global health challenges. Continued support from WOAH and its initiatives is imperative as we navigate the complexities of wildlife health on a global scale.

## Data availability statement

Publicly available datasets were analyzed in this study. This data can be found at: https://wahis.woah.org/#/home.

## Author contributions

LT: Conceptualization, Supervision, Validation, Writing – original draft, Writing – review & editing. CC: Writing – original draft, Writing – review & editing. LA: Writing – original draft, Writing – review & editing. SM: Writing – original draft, Writing – review & editing. DS: Writing – original draft, Writing – review & editing. JW: Writing – review & editing. PT: Conceptualization, Supervision, Validation, Writing – original draft, Writing – review & editing.
